# Effect of dietary supplement from mono‐culture fermentation of *Moringa oleifera* seeds by *Rhizopus stolonifer* on hematology and markers linked to hypercholesterolemia in rat model

**DOI:** 10.1002/fsn3.729

**Published:** 2018-08-14

**Authors:** Majekodunmi R. Adedayo, Jacob K. Akintunde, Alhassan Sani, Aline A. Boligon

**Affiliations:** ^1^ Department of Microbiology College of Pure and Applied Sciences Kwara State University Malete Nigeria; ^2^ Faculty of Science Department of Microbiology University of Ilorin Ilorin Nigeria; ^3^ Applied Biochemistry and Molecular Toxicology Research Group Department of Biochemistry College of Biological Sciences Federal University of Agriculture Abeokuta Nigeria; ^4^ Phytochemical Research Laboratories Department of Industrial Pharmacy Federal University of Santa Maria Santa Maria Brazil

**Keywords:** fermentation, hematology, lipid profile, mono‐culture, *Moringa oleifera*, rat, *Rhizopus stolonifer*

## Abstract

Mono‐culture fermentation by *Rhizopus stolonifer* could promote the healthiness of immune systems and cholesterol levels. Hence, we examined the effect of diet from mono‐culture fermentation of *Moringa oleifera* seeds by *R. stolonifer* (MCF‐MORS) on hematological parameters and fundamental indicators of hypercholesterolemia in rat. The animals were divided into six groups (*n *=* *6). Group 1 was placed on basal diet. Group II, III, IV and V were placed on a basal diets supplemented with 7.5%, 15%, 22.5% and 30%, respectively, of MCF‐MORS. Group VI was placed on basal diet fed with unfermented *M. oleifera* seeds (UF‐MOS). The experiment lasted for eight weeks. The results revealed 7.5% MCF‐MORS as better biological method to augment PCV, RBC and Hb count in animal model. Also, 7.5% and/or 15% MCF‐MORS demonstrated highest levels in centrophils, neutrophils and eosinophils, whereas the levels of lymphocytes, basophils and monocytes showed no significant difference. Similarly, 7.5% and 15% MCF‐MORS modulated LDL and HDL, respectively, better than UF‐MOS; but showing no difference in cholesterol level. MCF‐MORS also maintained architectural integrity of villi and splenocytes better than UF‐MOS. We therefore concluded that diet from MCF‐MORS at 7.5% and 15% modulates HDL, LDL, cholesterol and immune system‐related disorders better than UF‐MOS in rat model.

## 1 INTRODUCTION

*Moringa oleifera* (MO) is regarded as miracle plant due to its multiple uses. Virtually, all the parts of the plant are edible and utilized as traditional diets in many countries of the tropics and subtropics (Farooq & Umer, 2007). The stem bark, root, bark, fruit, flowers, leaves, seeds, and gum are widely used in India folk medicine (Compaoré et al., 2011), while the powdered seeds and roots are applied as spices and curries (Abdulkarim, Long, Lai, Muhammad, & Ghazali, 2005) and feed supplement for livestock. Also, recent studies described its nutritional and medicinal properties as a nonfood product (Anjorin, Ikokoh, & Okolo, 2010; Anwar, Latif, Ashraf, & Gilani, 2007). They also reported that the leaves contain both vitamins and essential minerals such as vitamin A, vitamin B, vitamin C, calcium, iron, potassium, essential amino acids, and high protein content (Anjorin et al., 2010).

Experimentally, some findings reported the efficacy of MO seeds against arsenic‐induced hepatocellular degeneration in female albino rats (Chattopadhyay et al., 2011). Also, *M. oleifera* showed a notable array of medicinal applications with high dietary value (Anwar et al., 2007). Anti‐inflammatory efficiency and hypotensive property of *M. oleifera* have been documented (Ndiaye et al., 2002). It was similarly demonstrated that *M. oleifera* leaves inhibited hyperlipidemia and hepatocytic disarrays when initiated by dietary iron shortage (Ndong, Uehara, Katsumata, Sato, & Suzuki, 2007), while its seed extract protected hepatic cells from necrotic damage and fibrosis in rat model (Fakurazi, Hairuszah, & Nanthini, 2008; Hamza, 2010). Further result also suggested that edible oil from MO seeds potentially protected rats in chemical‐induced hepatitis (Mansour et al., 2012). Another study similarly indicated that co‐administration of monoisoamyl dimercaptosuccinic acid (MDA) and MO seeds powder protected arsenic‐induced oxidative stress and metals distribution in mice (Mansour et al., 2012). Following these preventive effects of MO seed, recent study proved that fermentation of MO seeds increased the protein content, essential amino acid, and polyunsaturated fatty acid profiles with concomitant reduction in its anti‐nutrient compositions (Oluwole, Oluwole, & Oluwaseun, 2013).They then advocated that its fermented products should be consumed rather than the germinated and raw *M oleifera* seeds because of its high contents in essential minerals (Oluwole et al., 2013).

Fundamentally, fermentation is one of the biotechnological methods employed to potentiate the nutritional quality of legumes, cereals, and all form of seeds (Zhang, Xu, & Wang, 2007). Also, it is widely used for producing and preserving foods on local and industrial levels (Ndams, Tegbe, Ogundipe, & Sheyin, 2011). Strong evidence has shown that fermentation enhances nutritional value for several fermented products, especially yoghurt and wine (Zhao, Zhang, & Zhang, 2010). Recent finding reported that microorganisms have been found to be highly diverse in biochemistry, physiology, and nutritional modes (Vogel, Sarath, Saathoff, & Mitchell, 2011). But its selection for industrial application should be based on its ability to produce high yields of the desired product (Vogel et al., 2011; Zhang et al., 2007). Although, the nutritional value of fermented foods had long been recognized, but the scientific bases for many of the nutritional claims have been scantly studied (Kavanagh, 2005). It was reported that protein of plant source has more nutritional benefits than animal origin without health risks to livestock and humans (Melanson, MacLean, & Hill, 2009). Also, the fermentation of crop residues or seeds improves the digestibility of feedstuff (Yu, Guo, Zhang, Yan, & Xu, 2009) and the nutritional value particularly with the use of fungi to disrupt plant cell wall for promoting fermentable energy to the intestinal microbes. Specifically, mono‐culture fermentation of MO seeds by fungus to increase protein availability, digestibility, and reduction of its anti‐nutrient contents is still current (Nuha, Isam, & Elfadil, 2010) as there is little or no information on immuno‐protective action of fermented *M. oleifera* (FMO) seeds. However, we speculated that mono‐culture fermentation by *Rhizopus stolonifer* could promote the healthiness of immune systems and cholesterol levels in mammals. We also hypothesized whether dietary FMO seeds could modulate metabolic toxicity than dietary raw *M. oleifera* (RMO) in rattus novengicus model. For this reason, this study examined the effect of diet from mono‐culture fermentation of MO seeds by *R. stolonifer* on hematological parameters and markers linked to hypercholesterolemia in rat model.

## 2 MATERIALS AND METHODS

### 2.1 Sample selection and preparation

The fresh sample was collected from the premises of Nigerian Stored Products Research Institute (NSPRI) Kano, Nigeria. Authentication of the plant was carried out at the Herbarium section of the Department of Biology, University of Ilorin as *M. oleifera* with voucher number UIH 1011. *Moringa oleifera* pods were cracked open manually to release the dry seeds. The seeds were dried at 60°C in the oven to maintain the moisture content. Thereafter, seeds were powdered with an electrical grinder to particle size of about 2 mm and stored in air tight container for further use.

### 2.2 Quantification of compounds by HPLC‐DAD

Analysis of phenolic compounds by HPLC‐DAD Reverse phase chromatographic analyses was carried out under gradient conditions by the method of Boligon et al. (2015) and Reis et al. (2014).

### 2.3 Natural fermentation of *Moringa oleifera* powder

The fermentation of the powdered *M. Oleifera* seeds was performed according to the method described by Kayode and Sani (2008). Briefly, 5 g of powdered seeds was added to 5 ml of sterile distilled water. The mixture was stirred properly for a uniform mash to be obtained. The fermenter was covered, and the content was allowed to ferment at room temperature (28 ± 2°C) for 7 days. Also, the anti‐nutrients for both fermented and nonfermented MO seeds were determined. In addition, macro‐ and micro‐elements for both fermented and nonfermented MO seeds were determined by flame atomic absorption spectrophotometer.

### 2.4 Isolation of fungal organisms from naturally fermented *Moringa oleifera* seeds

Fungi were isolated from the naturally fermented seeds through serial dilution and pour plate methods using Potato Dextrose Agar (PDA) into which 10% streptomycin solution was added to inhibit bacteria growth. Culturing was performed in duplicates. The plates were incubated at room temperature (28 ± 2°C) for 48 hr. Isolated visible fungal colonies were further subcultured to obtain pure cultures. Morphological and microscopic analysis to identify the isolates were carried out and compared with literature (Stanley, Elizabeth, & Holt, 2001).The pure isolates were maintained on agar slant and kept in the refrigerator at 4°C for further use.

### 2.5 Macroscopic characterization of fungi isolates from *Moringa oleifera* seeds

The plates were examined macroscopically for morphological characteristics. Characteristics considered were color of the colony, production and release of pigment into the medium, rate of growth (fast or slow) during incubation, diameter of colony, spreading of the colony and the mycelia. Characteristics observed were in line with literature for the identification of the isolates (Stanley, 2001).

### 2.6 Microscopic characterization of fungi isolates from *Moringa oleifera* seeds

The isolates were observed in wet mount preparation under X10 and under X40 objective lens. They were observed before staining with cotton‐blue in Lactophenol and observed again under X10 and under X40 objective lens. Indexes of observation were hyphae length, type (sporangiophore or conidiophores), breath, size, color, presence or absence of septa, rising or spreading, branching type, spore shape, and texture of the cell wall (smooth or rough). The observations were identical with literature (Stanley, 2001).

### 2.7 Mono‐culture fermentation of *Moringa oleifera* seeds by *Rhizopus stolonifer* (MCF‐MORS)

*Rhizopus stolonifer* spore suspension of actively growing mid log phase culture was prepared according to the method described by Sani, Awe, and Akinyanju (1992). Briefly, an agar slant of four day old pure culture of *R. stolonifer* was used. Ten milliliters sterile distilled water was added to the slant and shaken to wash the spores. The spore suspension was counted using the Neubauer counting chamber. A spore suspension of about 5 × 10^4^ spore suspension/ml was used for inoculation. Exactly, 20 g of powdered *M. oleifera* seed sample was added into 250 ml Erlenmeyer flask, plugged with cotton wool, wrapped with aluminum foil, and sterilized in the autoclave at 121°C for 15 min. The sterile sample was mixed with 20 ml of sterile distilled water and stirred properly until uniform mashes were obtained. Thereafter, 2 ml of *R. stolonifer* mono‐culture suspension was used as fermentation starter to inoculate *M. oleifera* sample in the fermenter. The mixture was allowed to ferment for 72 hr at 28 ± 2°C (Kayode & Sani, 2008; Lawal, Iyayi, & Aderemi, 2005). Fermented sample was dried at 60°C in the oven for 4 hr. The residue was used for diet formulation, and the proximate composition was analyzed.

### 2.8 Diet formulation

Experimental diets supplemented with four varied doses (7.5%, 15%, 22.5% and 30%) of mono‐culture fermentation of *M. oleifera* seeds by *R. stolonifer* (MCF‐MORS) for 72 hr were formulated according to a modified method of Yusuf, Bamgbose, Oso, Fafiolu, and Oni (2008) (Table 1). Soybean was used as the source of protein. This was prepared by adding 30%, 22.5%, 15%, and 7.5% of soybean meal to obtain 100% formulation (Table 2). Thereafter, the proximate quantity of the diet used to feed the animals in each group was measured as reported in Table 2. The unfermented *M. oleifera* seed was designated as UF‐MOS.

**Table 1:** Diet formulation for basal and supplemented diets in control and test groups

Treatment	Group 1	Group 2	Group 3	Group 4	Group 5	Group 6
Corn meal	50.6	50.6	50.6	50.6	50.6	50.6
Cellulose	3.0	3.0	3.0	3.0	3.0	3.0
Sucrose	8.0	8.0	8.0	8.0	8.0	8.0
Premix	3.0	3.0	3.0	3.0	3.0	3.0
D‐methionine	0.4	0.4	0.4	0.4	0.4	0.4
Oil	5.0	5.0	5.0	5.0	5.0	5.0
Soya bean meal	30.0	22.5	15.0	7.5	—	—
MCF‐MORS	—	7.5	15.0	22.5	30.0	—
UF‐MOS	—	—	—	—	—	30.0
Total (g)	100	100	100	100	100	100

Notes

MCF‐MORS: Diet from Mono‐culture fermentation of MO seeds by *Rhizopus stolonifer*; UF‐MOS: Diet from unfermented MO seeds.

The vitamin premix (mg or IU/g) has the following composition; 3200 IU vitamin A, 600 IU vitamin D3, 2.8 mg vitamin E, 0.6 mg vitamin K3, 0.8 mg vitamin B1, 1 mg vitamin B2, 6 mg niacin, 2.2 mg pantothenic acid, 0.8 mg vitamin B6, 0.004 mg vitamin B12, 0.2 mg folic acid, 0.1 mg biotin H2, 70 mg choline chloride, 0.08 mg cobalt, 1.2 mg copper, 0.4 mg iodine, 8.4 mg iron, 16 mg manganese, 0.08 mg selenium, 12.4 mg zinc, 0.5 mg antioxidant. Group 1: serve as the control group placed on a basal diet; Group 2: serve as the group placed on a basal diet supplemented with 7.5% of mono‐culture fermented pulverized seeds of *Moringa oleifera* Group 3: serve as the group placed on a basal diet supplemented with 15% of mono‐culture fermented pulverized seeds of *M. oleifera*; Group 4: serve as the group placed on a basal diet supplemented with 22.5% of mono‐culture fermented pulverized seeds of *M. oleifera*; Group 5: serve as the group placed on a basal diet supplemented with 30% of mono‐culture fermented pulverized seeds of *M. oleifera*; Group 6: (Negative control) serve as the normal group placed on a basal diet supplemented with 30% of unfermented pulverized seeds of *M. oleifera*.

**Table 2:** Proximate composition (%) of the diets used to feed the control and test groups

Group	MC	DM	CP	CF	CFB	TA	N
Group 1	7.25	92.75	27.92	6.94	3.95	4.74	49.10
Group 2	7.65	92.35	27.95	8.45	3.81	3.27	48.87
Group 3	7.40	92.60	27.60	8.40	3.90	3.39	49.31
Group 4	7.33	92.67	27.20	8.73	3.64	3.50	49.60
Group 5	7.15	92.85	26.87	8.95	4.02	3.57	49.44
Group 6	8.02	91.98	24.00	10.63	3.75	3.05	36.51

Note

MC: moisture content; DM: dry matter; CP: crude protein; CF: crude fat; CFB: crude fiber; TA: total ash; N: Nitrogen.

### 2.9 Animal handling

Weaned Wistar rats (43.3 ± 2.5 g) from the Central Animal House of the University of Ilorin, Ilorin were used in this experiment. The animals were maintained at a constant temperature (22 ± 2°C) on a 12‐hr light/dark cycle with free access to food and water.

### 2.10 Experimental protocol

The weaned rats were acclimatized for 2 weeks and randomly divided into six groups of six animals each (*n *=* *6). Group 1 serve as the control group placed on a basal diet; Group 2 was placed on a basal diet supplemented with 7.5% MCF‐MORS. Group 3 was placed on a basal diet supplemented with 15% MCF‐MORS. Group 4 was placed on a basal diet supplemented with 22.5% MCF‐MORS. Group 5 was placed on a basal diet supplemented with 30% MCF‐MORS. Group 6 (Negative control) was placed on a basal diet supplemented with 30% UF‐MOS. The experiment lasted for 8 weeks. The animals were fasted overnight, weighed and euthanized 24 hr after the last treatment, and blood was collected by cardiac puncture. The blood was allowed to clot and centrifuged at low speed (3,000 *g*) at room temperature for 15 min. The supernatant (plasma) was removed and used for the determination of biochemical parameters.

### 2.11 Chemicals and reagents

All the kits used for the bioassay were sourced from Randox Laboratories Ltd. (Crumlin, Dublin, Northern Ireland, UK). All other reagents used were in the purest form (analytical grade) available commercially.

### 2.12 Hematological and immunological estimation

Hemoglobin concentration (Hb %) was measured by Drabkin's colorimetric method (cyanomethemolgobin formation), and packed cell volume (PCV) was estimated by scale of microhematocrit reader (Duncan, Prasse, & Mahaffey, 1994). The concentration of red blood cells (RBC) and white blood cells (WBC) was measured in the given blood sample and was expressed as volume of cells (Coles, 1986). Additionally, neutrophils, lymphocytes, monocytes, eosinophils, basophils, and centrophils were determined using conventional method of Dacie and Lewis (1995).

### 2.13 Determination of HDL, LDL, and cholesterol

The key lipid profiles: high‐density lipoprotein (HDL), low‐density lipoprotein (LDL), and cholesterol were measured using commercially available kits (Randox Laboratories Kits, St Louis, MO, USA) according to the manufacturer's guideline.

### 2.14 Histological examination

Small intestine and spleen specimen were fixed in 10% neutral buffered formalin, embedded in paraffin, and sectioned. After deparaffinization and dehydration, the paraffin blocks were stained with hematoxylin and eosin for microscopic examination.

### 2.15 Statistical analysis

The data in each group were expressed as mean ± standard deviation. A one‐way analysis of variance (ANOVA) was used to analyze the results and Duncan multiple test was used for the post hoc (Zar, 1984). Statistical package for Social Science (SPSS) 17.0 for windows was used for the analysis, and the least significance difference (LSD) was accepted at *p *<* *0.05.

## 3 RESULTS AND DISCUSSION

The use of fungi and/or their enzymes that metabolize lignocelluloses had been reported as a potential biological treatment to improve the nutritional significance (Abdolhamid, Mohamad, Mohsen, & Mohsen, 2014). Also, it was recently explicated that soaking, sprouting, and fermentation have beneficial effects as biological methods for food optimization (Emtenan et al., 2012). Hence, this present study examines the effects of dietary supplement from mono‐culture fermentation of MO seeds by *R. stolonifer* on hematological parameters and markers linked to hypercholesterolemia in experimental rats. With the exclusion of moisture content, crude fat, crude fiber, and carbohydrate, the proximate parameters such as total ash and crude protein in MCF‐MORS were significantly (*p *<* *0.05) higher than the UF‐MOS particularly at 48 and 72 hr (Table 3). This suggests that mono‐culture fermentation by *R. stolonifer* could increase the protein content of MO seeds. The study was in line with recent finding that natural fermentation elevates protein levels in MO seeds (Oluwole et al., 2013) and *Pakia biglobosa* (Compaoré et al., 2011). Also, the fermented MO seeds by *R. stolonifer* decreased the levels of anti‐nutrients such as saponin, oxalate, tannin, and phytate (Table 4) in relation to nonfermented MO seeds. This indicates that UF‐MOS contain high levels of anti‐nutrients. Studies had reported that excess intake of anti‐nutrients particularly saponin, tannin, oxalate, and lectin could cause severe intestinal damage disrupting digestion and nutrients shortages (Fereidoon, 2014; Habtamu & Negussie, 2014). These secondary compounds could trigger IgG and IgM antibodies to elicit abnormal immune responses and concurrently bind to erythrocytes to produce hemagglutination and anemia (Sano & Ogawa, 2014). Furthermore, essential elements such as potassium, magnesium, iron, and copper were significantly (*p *<* *0.05) increased during fermentation by *R. stolonifer* with concomitant reduction in the levels of sodium, calcium, manganese, zinc, cadmium, and lead (Table 5). The reduction may be linked to the complex formation of oxalate with these elements (Na^+^, Ca^2+^, Mn^2+^, Zn^2+^, Cd^2+^, and Pb^2+^) (Baoquan, Timothy, Ballav, & George, 2015) during mono‐culture fermentation by *R. stolonifer*. This therefore suggests that mono‐culture fermentation by *R. stolonifer* is one of the basic techniques to attenuate the levels of anti‐nutrients and some excess macro‐/micro‐nutrients in MO seeds. It was previously shown that Cd could cause severe damage to erythrocytes and immune cells in man (Rachel, Thomas, Miquel, Ingvar, & Domenico, 2013). Cd^2+^ metabolite in the blood of occupationally exposed workers was also connected to erythrocyte cancer and lymphocyte impairment (Amati et al., 2008). It has similarly been reported that Ca overload had a correlative effect on hyperlipidemia, cardiovascular dysfunction, and hypertension when triggered by oxidative stress (Nisha, Keshav, & Kalpana, 2016). Further report also showed that exposure to Pb significantly (*p *<* *0.05) decreased human hemoglobin (Hb) with evidence of prolonged hemoglobinopathies (Bernard & Franklin, 2013). Lastly, exposure to surplus inorganic sodium could initiate high blood pressure with severe thrombosis (Kalaitzis, Pasadakis, Bantis, Giannakopoulos, & Touloupidis, 2010). However, these abnormalities may not be propounded in vivo following the mono‐culture fermentation of MO seeds by *R. stolonifer*.

**Table 3:** Effect of mono‐culture fermentation *of Moringa oleifera* seeds by *Rhizopus stolonifer* on proximate composition (g/100 g) between 0 and 72 hr

Fermentation period (hr)	MC	TA	CP	CF	CFB	CHO
0	7.81 ± 1.40^a^	2.35 ± 0.06^a^	21.10 ± 1.62^a^	27.44 ± 0.25^a^	4.39 ± 0.64^a^	27.44 ± 8.13^a^
24	5.93 ± 1.28^a^	2.34 ± 0.06^a^	32.34 ± 0.41^b^	26.62 ± 0.23^a^	4.49 ± 0.75^a^	26.62 ± 4.79^a^
48	6.10 ± 0.69^a^	2.58 ± 0.13^b^	32.98 ± 0.48^b^	25.03 ± 2.45^a^	4.63 ± 0.95^a^	25.03 ± 2.05^a^
72	5.38 ± 1.16^a^	3.15 ± 0.00^b^	31.96 ± 0.00^b^	30.43 ± 0.00^ab^	5.28 ± 0.00^a^	30.43 ± 0.00^ab^

Notes

MC: moisture content; DM: dry matter; CP: crude protein; CF: crude fat; CFB: crude fiber; TA: total ash; N: Nitrogen.

Data are presented as mean ± *SD* (*n *=* *2). Bars with different letters on the same column are statistically different (*p *<* *0.05).

**Table 4:** Effect of mono‐culture fermentation of *Moringa oleifera* seed by *Rhizopus stolonifer* on anti‐nutrients composition

Phytochemical	UF‐MOS (mg/g)	MCF‐MORS (mg/g)
Saponin	8.58	1.86
Oxalate	4.86	1.88
Tannins	3.37	0.26
Phytate	2.96	0.35

**Table 5:** Effect of mono‐culture fermentation by *Rhizopus stolonifer* on some macro and micro‐elements using *Moringa oleifera* seed as substrate

Element	UF‐MOS (mg/100 g)	MCF‐MORS (mg/100 g)
Potassium	97.00 ± 0.0^a^	120.8 ± 0.03^b^
Sodium	190.00 ± 0.0^b^	89.7 ± 0.70^a^
Calcium	758.00 ± 0.0^b^	210.6 ± 2.1^a^
Magnesium	308.00 ± 0.0^a^	1690.0 ± 2.1^b^
Manganese	28.00 ± 0.0^b^	15.12 ± 1.4^a^
Iron	94.00 ± 0.0^a^	104.95 ± 2.3^b^
Zinc	67.30 ± 0.0^b^	37.67 ± 0.35^a^
Copper	5.00 ± 0.0^a^	8.18 ± 0.00^b^
Cadmium	2.54 ± 0.00^b^	0.50 ± 0.34^a^
Lead	8.20 ± 0.0^b^	0.00 ± 0.00^a^

Note

Data are presented as mean± *SD* (*n *=* *2). Bars with different letters on the same row are statistically different (*p *<* *0.05).

**Figure 1:**
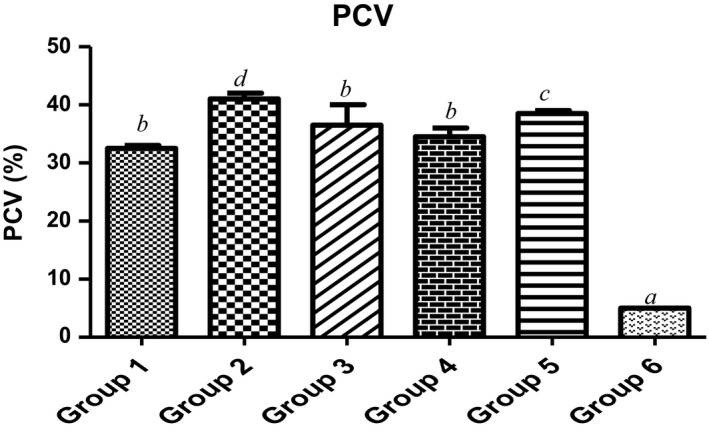
Effect of MCF‐MORS and UF‐MOS on Part Cell Volume (PCV) in treated rat. Data are presented as mean ± *SD* (*n *=* *6). Bars with different letters are statistically different

The percentage body weight gain of the group of animals treated with UF‐MOS was lower than MCF‐MORS (7.5%, 15%, 22.5% and 30%) and the control (Table 6). This study explains that the body weight may depend on PCV amounts, erythrocyte counts, and immune systems. Our opinion corroborates the former result which reported that body weight and leukocyte counts were significantly (*p *<* *0.05) lowered in the anemic piglets than the normal group (Pu et al., 2015). Also, the blood quantity and quality of packed cell volume (PCV) were significantly (*p *<* *0.05) elevated in animals fed with MCF‐MORS when compared to UF‐MOS group (Figure 1). Specifically, PCV count was considerably (*p *<* *0.05) lowered by 84.6% in group of animal supplemented with UF‐MOS in relation to the corresponding control (Figure 1). The significant increase of PCV count (i.e., MCF‐MORS) indicates optimum production in total blood counts by the bone marrow due to the high contents of crude protein and magnesium particularly dietary iron from plant origin. This agrees with the previous study which reported that abnormal or irregular supply of nutrient‐rich iron caused the red blood cells to be malformed or die off at a faster rate than the body can replace them, leading to declined PCV counts (Pu et al., 2015). Essentially, reduced iron is the nutrient commonly associated with anemia because the body uses iron to make the hemoglobin to transport oxygen in the blood cells. However, increased PCV counts and the regeneration of red blood cells had been connected to the wellness of the bone marrow cells potentiated by dietary magnesium, iron, and protein (Gregory, 2006).

**Table 6:** Effect of MCF‐MORS and UF‐MOS o n body weight growth in rat

Group	Initial body Weight (g)	Final body Weight (g)	Weight gain
Group 1	43.3 ± 2.5^a^	92.8 ± 3.5^bc^	49.5 ± 1.0^ab^
Group 2	43.5 ± 3.5^a^	94.0 ± 3.0^ab^	51.5 ± 6.5^b^
Group 3	43.5 ± 2.5^a^	105.5 ± 0.5^bc^	58.5 ± 2.0^c^
Group 4	45.0 ± 5.0^a^	108.0 ± 5.0^c^	65.6 ± 2.5^c^
Group 5	45.0 ± 10.0^a^	94.0 ± 1.0^abc^	49.0 ± 11.0^ab^
Group 6	45.5 ± 2.5^a^	76.0 ± 5.0^a^	30.5 ± 2.5^a^

Note

Values represent mean ± *SD*,* n *=* *6; Values with different superscript on the same column are significantly (*p *<* *0.05) different.

**Figure 2:**
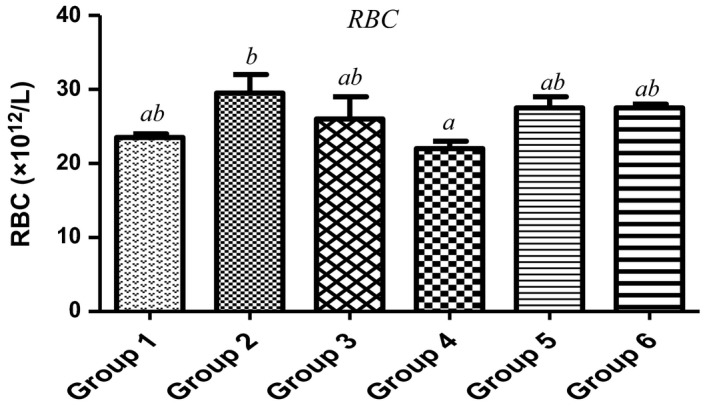
Effect of MCF‐MORS and UF‐MOS on Red Blood Cells (RBC) in treated rat. Data are presented as mean ± *SD* (*n *=* *6). Bars with different letters are statistically different

**Figure 3:**
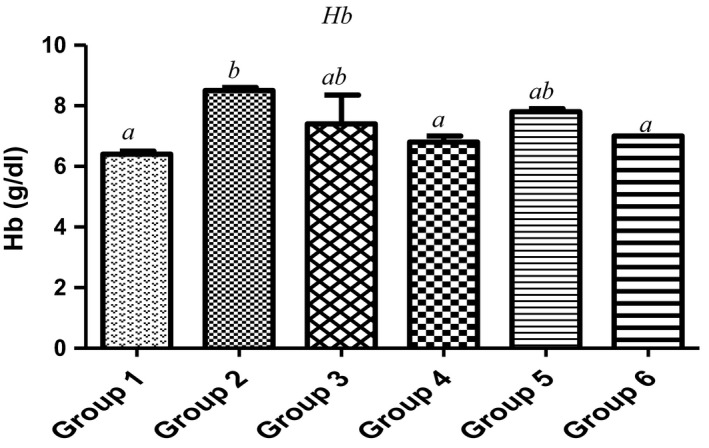
Effect of MCF‐MORS and UF‐MOS on Hemoglobin (Hb) in treated rat. Data are presented as mean ± *SD* (*n *=* *6). Bars with different letters are statistically different

**Figure 4:**
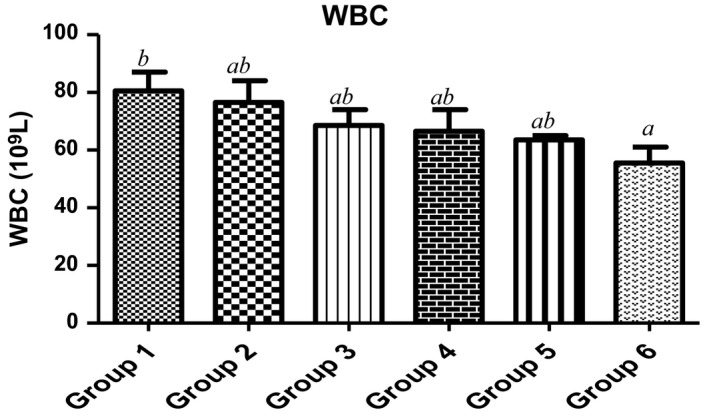
Effect of MCF‐MORS and UF‐MOS on White Blood Cells (WBC) in treated rat. Data are presented as mean ± *SD* (*n *=* *6). Bars with different letters are statistically different

Red blood cells (RBC) are made in the spongy center of the bone marrow which contains hemoglobin (Hb). Their deficiencies are one of the key risk factors of multiple cancers such as leukemia, lymphoma, and multiple myeloma (Friedenberg et al., 2016), all these resulting in anemia. As observed in Figure 2, with the exclusion of 7.5%, the animals fed with 15%, 22.5%, 30% of MCF‐MORS showed no significant (*p *<* *0.05) difference in the level of RBC when compared to the control and UF‐MOS. Similar trends were observed in the levels of Hb (Figure 3). This suggests that MCF‐MORS was more efficacious at lower dose than higher doses during the production of RBC and Hb in animal model. The reduction in RBC and Hb levels at higher doses hypothesized potent competitive interaction among inorganic substances particularly Ca^2+^, Mn^2+^, Zn^2+^, Cd^2+^, and Pb^2+^. Previous studies had shown that hematotoxic effects could be traced to additive, competitive, or antagonistic interactions of macro‐nutrients overload, all of which eventually interrupts hematopoiesis (Akintunde, Oboh, & Akindahunsi, 2015; Kaustav, Melanie, Abishek, David, & Matthew, 2016; Venkata, Zeeshan, Luqman, & Saif, 2015). Moreover, the reduction in the levels of RBC and Hb at higher doses may be linked to the remarkable dietary depletion of Mn^2+^ and Zn^2+^ levels by MCF‐MORS. Study has shown that Mn^2+^ and Zn^2+^ could be incorporated in human diets if RBC and Hb are depleted and they are also redox‐active elements essential as catalytically active cofactors in enzymes and structurally stabilizing protein production as well as enhancing immune systems (Friedenberg et al., 2016; James & Eric, 2013). Additionally, animal fed with 7.5%, 15%, 22.5%, and 30% MCF‐MORS showed no significant (*p *<* *0.05) difference in WBC count in relation to the control (Figure 4), whereas animals fed with UF‐MOS were significantly (*p *<* *0.05) reduced the level of WBC when compared to corresponding control (Figure 6). This depicts that the dietary inclusion of MCF‐MORS may boost the immune system particularly leukocytes (WBC) in animal model better than nonfermented MO seeds. This finding also speculated that dietary inclusion of MCF‐MORS would cause noticeable immune phagocytic actions and inflammatory defenses than UF‐MOS, suggesting its potent usefulness in clinical and signaling hematologic wellness (Burchi, Fanzo, & Frison, 2011; Vikash, Suvra, Debtanu, Lokesh, & Kundan, 2013). This observation corroborated the recent finding which reported that diets containing no or little anti‐nutrients such as oxalates, saponins, tannins, phytates, and lectins with tolerable proportions of essential minerals possess a greater potential to fight infections (James & Eric, 2013) and promote the immune cells against pathogens, damaged cells, cancerous cells, and abnormal stem cell development (Friedenberg et al., 2016; Kaustav et al., 2016). However, we proposed that MO seeds fermented by fungus known as *R. stolonifer* reduced possible risk of immune suppression and leukemia than nonfermented.

**Figure 5:**
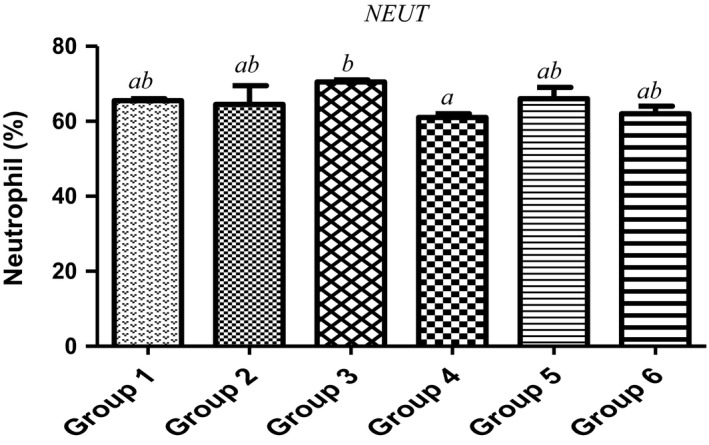
Effect of MCF‐MORS and UF‐MOS on Neutrophils in treated rat. Data are presented as mean ± *SD* (*n *=* *6). Bars with different letters are statistically different

**Figure 6:**
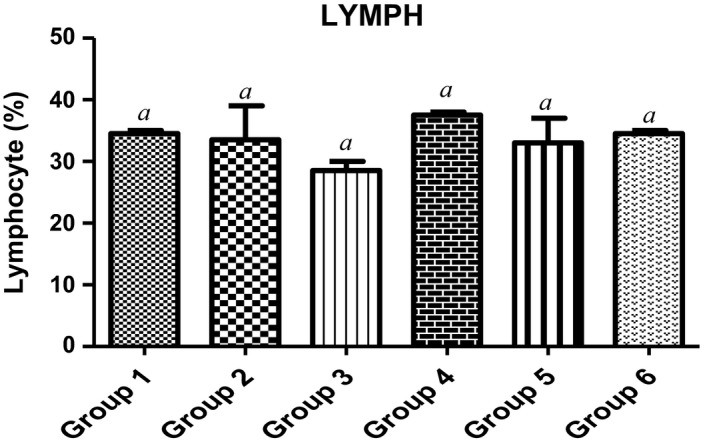
Effect of MCF‐MORS and UF‐MOS on lymphocytes in treated rat. Data are presented as mean ± *SD* (*n *=* *6). Bars with different letters are statistically different

**Figure 7:**
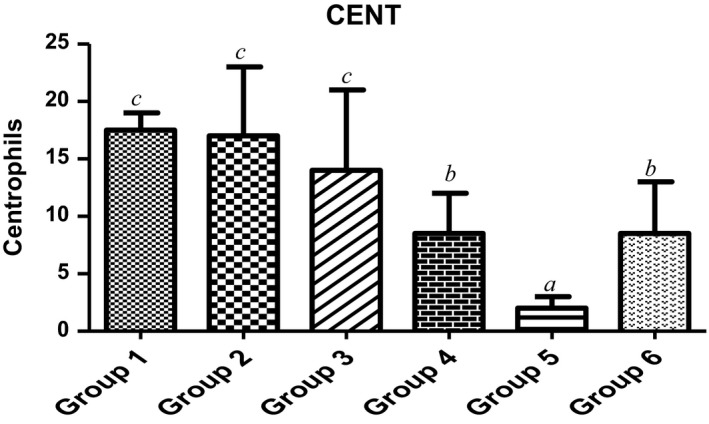
Effect of MCF‐MORS and UF‐MOS on Centrophils in treated rat. Data are presented as mean ± *SD* (*n *=* *6). Bars with different letters are statistically different

**Figure 8:**
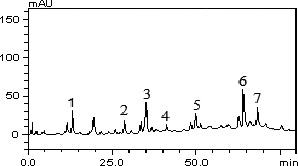
Representative high performance liquid chromatography profile of black seed aqueous extract. Gallic acid (peak 1), caffeic acid (peak 2), *p*‐coumaric acid (peak 3), Rutin (peak 4), quercetin (peak 5), luteolin (peak 6), and apigenin (peak 7)

Furthermore, lymphocytes, neutrophils and centrophils are the subtypes of white blood cells in a vertebrates’ immune system. As shown in Figures 5 and 6, animals fed with varying doses of MCF‐MORS showed no significant (*p *<* *0.05) difference in the levels of neutrophils and lymphocytes in all treated groups, whereas MCF‐MORS remarkably (*p *<* *0.05) hiked the level of centrophils particularly at 7.5% and 15% doses when compared to UF‐MOS group (Figure 7). This finding suggests that both MCF‐MORS and UF‐MOS groups function identically as cell mediated and cytotoxic innate/adaptive immunity. The diets have similar potency to avert neutropenia by potentially creating antibodies (T and B lymphocytes) which vehemently fight against bacteria, viruses, and other possible harmful invaders. The similarity in their efficacies could be traced to abundant levels of micronutrients and the phenolic compounds particularly luteolin, p‐coumaric acid, and gallic acid (Figure 8 and Table 7). This is because studies had documented that flavonoids and phenolic acids act as antioxidants to maintain strong T and B lymphocytes (phagocytes) and reduce vulnerability of the body to infectious diseases as well as preventing angiogenesis in cancer cells (Burchi et al., 2011; Tanya, Xavier, & Clifford, 2014). Centrophils produced from pluripotent hemopoietic stem cells further suggest that MCF‐MORS provokes the production of growth factors (granulocyte‐colony‐stimulating factor [G‐CSF], granulocyte‐macrophage‐colony stimulating factor [GM‐CSF], interleukin 3 and macrophage‐colony‐stimulating factor [M‐CSF]) better than UF‐MOS; as such, prolonged the survival of T‐lymphocytes and natural killer cells (NKs) (Mendy, Yonglian, & Yang‐Xin, 2009) by upregulating the immune response via cytokine release (Jamila, Bharti, & Kanti, 2003; Michael, Sarah, Neda, Wood, & Shayan, 2013). Moreover, recent study had reported that eosinophils possess substantial pro‐inflammatory and cytotoxic activity and responsible for the pathogenesis of various disease processes (Ilesanmi et al., 2016). However, eosinophils were not produced in animals fed with 7.5%, 15%, and 22.5% MCF‐MORS as well as the control group (Table 8), signifying its nontoxic effect. Conversely, animal fed with UF‐MOS significantly (*p *<* *0.05) increased the level of eosinophils (Table 8). This hike level may be attributed to some quantities of heavy metals in UF‐MOS particularly Cd^2+^ and Pb^2+^. Recent study had implicated high level of eosinophils in the blood among workers exposed to Pb^2+^ (Patricia & Marc, 2013). Basophils were not detected in all the groups (Table 8). This suggests that both MCF‐MORS and UF‐MOS have the ability of inhibiting asthma, allergic rhinitis, and anaphylaxis (Gianni, Francesco, Gilda, Arturo, & Francescopaolo, 2014). Lastly, groups of animals fed with 7.5%, 15%, and 22.5% MCF‐MORS showed no significant (*p *<* *0.05) increase in the level of monocytes when compared to the control and UF‐MOS group (Table 8). This indicates that level of monocytes produced is still within the tolerable limit for mammals. Our finding supports the earlier finding which stated that uncontrollable high content of monocyte counts greater than 0·9 × 10^9^/L was associated with the development of monocytosis, pericarditis, or joint effusions and ascites (Papageorgiou et al., 2013).

**Table 7:** Composition of phenolic compounds in MO seeds

Compounds	*M. oleifera* (mg/g)	LOD (μg/ml)	LOQ (μg/ml)
Gallic acid	2.46 ± 0.01^a^	0.021	0.068
Caffeic acid	1.53 ± 0.01^b^	0.018	0.059
p‐coumaric acid	3.11 ± 0.03c	0.009	0.030
Rutin	0.97 ± 0.02^d^	0.025	0.082
Quercetin	1.45 ± 0.01^b^	0.009	0.030
Luteolin	6.05 ± 0.04^e^	0.027	0.089
Apigenin	1.49 ± 0.03^b^	0.015	0.049

Note

The results are expressed as mean ± SEM of three determinations. Comparing various groups, different letters indicate statistically significant findings.

**Table 8:** Effect of MCF‐MORS and UF‐MOS on eosinophil, monocytes, and basophils in treated rat

Marker	Eosinophil (%)	Monocytes (%)	Basophils (%)
Group 1	0.00^a^	0.00^a^	0.00
Group 2	0.00^a^	2.5 ± 0.5^a^	0.00
Group 3	0.00^a^	1.00^a^	0.00
Group 4	0.00^a^	1.50^a^	0.00
Group 5	1.00^a^	0.00^a^	0.00
Group 6	3.5 ± 0.5^b^	0.00^a^	0.00

Note

Data are presented as mean ± *SD* (*n *=* *6). Bars with different letters are statistically different.

**Figure 9:**
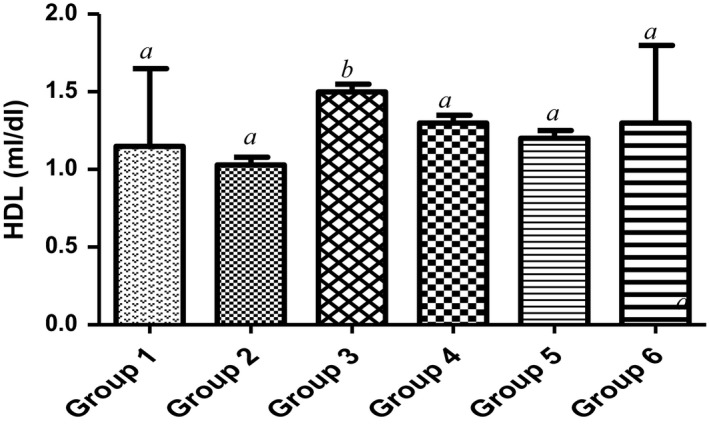
Effect of MCF‐MORS and UF‐MOS on high‐density lipoproteins (HDL) in treated rat. Data are presented as mean ± *SD* (*n *=* *6). Bars with different letters are statistically different

**Figure 10:**
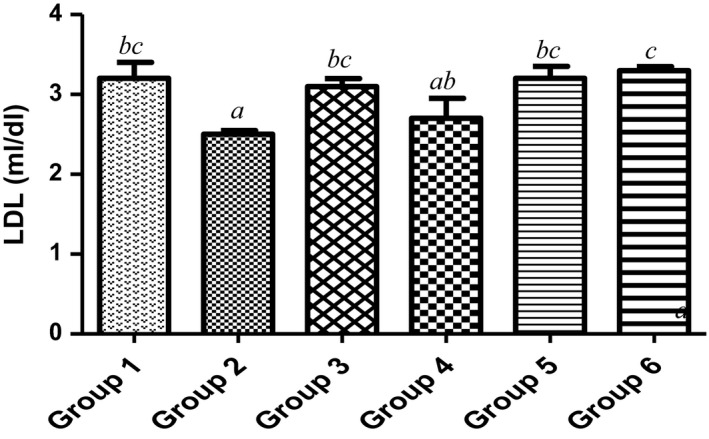
Effect of MCF‐MORS and UF‐MOS on low‐density lipoproteins (LDL) in treated rat. Data are presented as mean ± *SD* (*n *=* *6). Bars with different letters are statistically different

**Figure 11:**
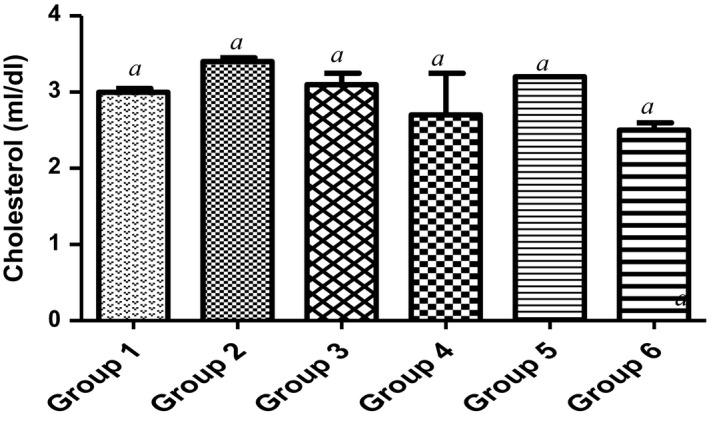
Effect of MCF‐MORS and UF‐MOS on cholesterol in treated rat. Data are presented as mean ± *SD* (*n *=* *6). Bars with different letters are statistically different

Countless public health investigations have linked elevated concentration of total cholesterol or LDL‐cholesterol in plasma to atherosclerosis (Yung‐Chih, Alex, Tin, Alex, & Karlheinz, 2016). Firstly, high‐density lipoprotein (HDL) of the animal dietetically fed with 15% MCF‐MORS significantly (*p *<* *0.05) increased plasma HDL in relation to animals treated with UF‐MOS (Figure 9) and the control. This suggests that 15% of dietary mono‐culture fermentation by *R. stolonifer* for 72 hr showed the best potency to picks up excess cholesterol in the blood to inhibit hyperlipidemia and complications of atherosclerosis (Mattar & Obeid, 2009; Shanmugma, Román‐Rego, Ong, & Kaski, 2010). Secondly, low‐density lipoprotein (LDL) was significantly (*p *<* *0.05) depleted in animal supplemented with 7.5% of MCF‐MORS when compared to UF‐MOS and the control (Figure 10). This result indicated that 7.5% mono‐culture fermentation for 72 hr of MO by *R. stolonifer* showed the most therapy to prevent the build‐up of (bad) LDL‐cholesterol within the walls of the blood vessels. Lastly, animals fed with MCF‐MORS in all treated groups showed no significant (*p *<* *0.05) difference in cholesterol levels (Figure 11). No significant difference advocates that MCF‐MORS and UF‐MOS correspondingly act as hypolipidemia, hypolipoproteinmia, and hypocholesterolemia agents. This was corroborated by the previous finding which reported that crude extract of MO lowered the principal markers linked with coronary artery disease and acute myocardial infarction in high‐fat diet rats (UNdong et al., 2007). Also, recent studies showed that MO seeds elevated red blood cells and immune system in cholesterol high fed rat (Frank, Henrietta, Ifeanyi, & Chizoma, 2014; Ochuko, Osaretin, Ebuehi, Muhammad, & Gloria, 2013).

**Figure 12:**
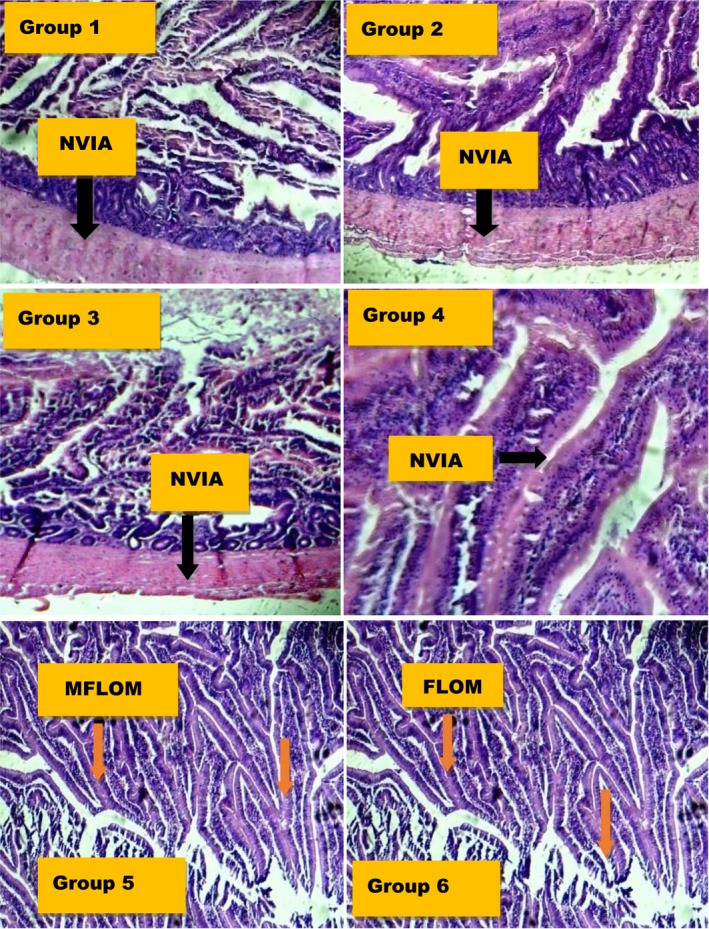
(Groups 1‐6) Effect of MCF‐MORS and UF‐MOS on small intestine of treated rat. (Group 1) showed normal and visible intestinal architecture (NVIA), that is, the villi were intact and normal architectural structures of the intestinal membrane were observed (Group 2) showed normal and visible intestinal architecture (NVIA) (Group 3) depicted normal and visible intestinal architecture (NVIA) (Group 4) revealed intact and normal architectural structures (NVIA) of the membrane and the villi were not affected. (Group 5) showed mild focal loss of the overlaying mucosa (MFLOM) (Group 6) showed focal loss of the overlaying mucosa (FLOM)

**Figure 13:**
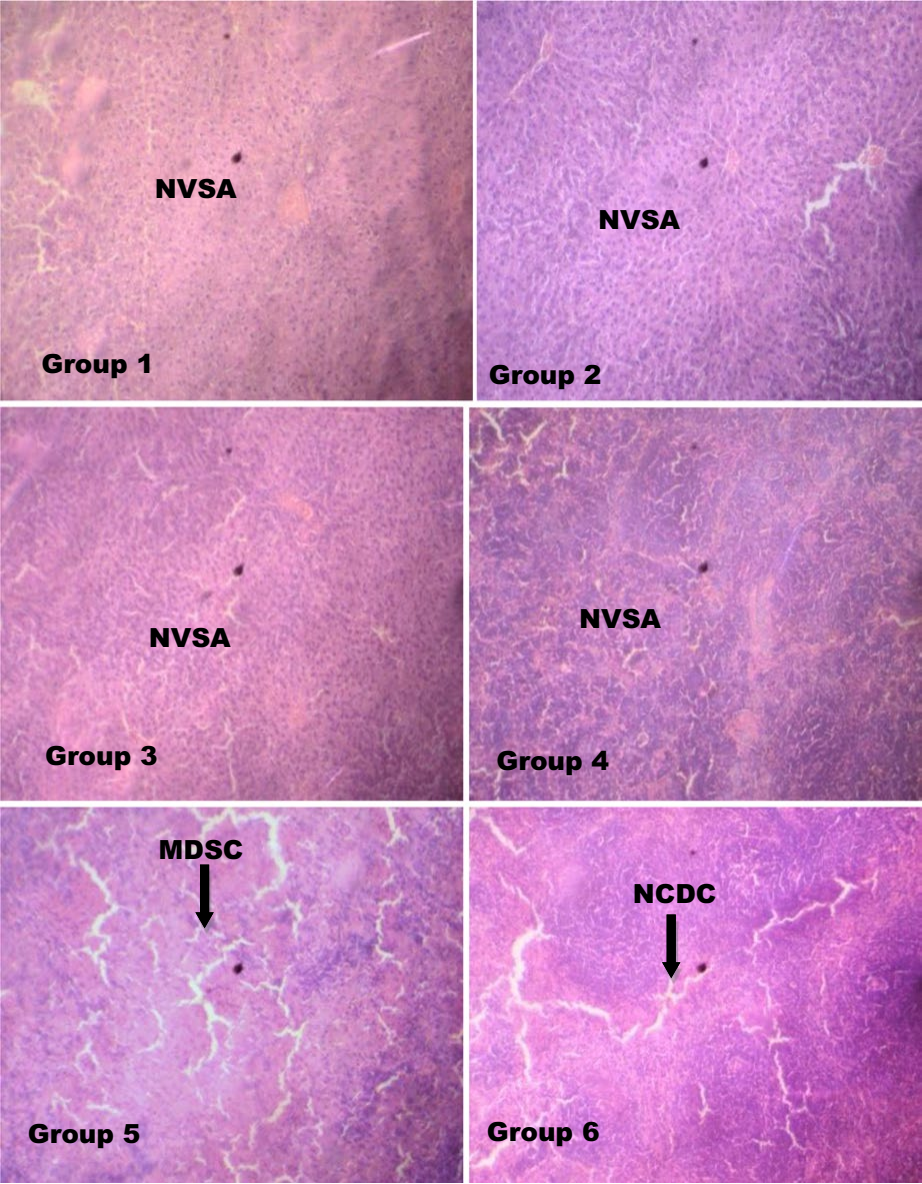
(Groups 1‐6) Effect of MCF‐MORS and UF‐MOS on spleen of treated rat. (Group 1) showed normal and visible spleen architecture (NVSA) (Group 2) showed normal and visible spleen architecture (NVSA) (Group 3) depicted normal and visible spleen architecture (NVSA) (Group 4) revealed normal spleen architectural structures (NVSA) (Group 5) showed mild degeneration of spleen cells (MDSC) (Group 6) showed nonchromic and distorted cells (NCDC)

Histopathologically, as depicted in Figure 12, experimental rats supplemented with dietary MCF‐MORS revealed no visible lesions, that is, normal architectural structures of the membrane and the villi; while there was concomitant manifestation of mild focal loss of the overlaying mucosa in animal supplemented with UF‐MOS. This detection is line with recent development which reported that the disruption and loss of villi or overlaying mucosa play an important function in pathogenesis of the small intestine resulting into accumulation of cholesterol (cholesterolemia) in the blood and complicated atherosclerosis (Shanmugma et al., 2010). Additionally, the spleen synthesizes antibodies in its white pulp, destroys old, and damaged cells. It responds actively to immune systems through humoral and cell‐mediated pathways. Hence, spleen dysfunctions such as splenomegaly, blood‐based leukemias, and asplenia have been conventionally identified (Shanmugma et al., 2010). In the present study, animal fed with MCF‐MORS showed no visible lesions to the spleen cells, that is, normal visible splenocytes (Figure 13). This asserts a better physiological wellness of T and B lymphocytes, granulocytes, dendritic cells, macrophages, and other immune cells (Friedenberg et al., 2016) than UF‐MOS.

## 4 CONCLUSION

This study discovered 7.5% MCF‐MORS as better biological method to augment PCV count, RBC count, and Hb in animal model. Also, 7.5% and/or 15% MCF‐MORS demonstrated highest levels in centrophils, neutrophils, and eosinophils, whereas the levels of lymphocytes, basophils, and monocytes showed no significant difference. Similarly, 7.5% and 15% MCF‐MORS modulated LDL and HDL, respectively, better than UF‐MOS; but showing no difference in cholesterol levels. MCF‐MORS also maintained architectural integrity of villi and splenocytes better than UF‐MOS. We therefore concluded that diet from MCF‐MORS for 72 hr at 7.5% and 15% modulates HDL, LDL, cholesterol, and immune system‐related disorders better than UF‐MOS in rat model.

## ETHICAL STATEMENTS

All the animals received humane care according to the criteria outlined in the Guide for the Care and Use of Laboratory Animals prepared by the National Academy of Science and published by the National Institute of Health. Ethic regulations have been followed in accordance with National and institutional guidelines for the protection of animal welfare during experiments. The study's protocols and procedures were ethically reviewed and approved by the ethical committee of the University of Ilorin—Nigeria by the number UIH 1011.

## CONFLICT OF INTEREST

The author(s) declared no potential conflicts of interest with respect to the research, authorship, and/or publication of this article.
